# The search for the Holy Grail: autoantigenic targets in primary sclerosing cholangitis associated with disease phenotype and neoplasia

**DOI:** 10.1186/s13317-020-00129-x

**Published:** 2020-03-16

**Authors:** Steffi Lopens, Marcin Krawczyk, Maria Papp, Piotr Milkiewicz, Peter Schierack, Yudong Liu, Ewa Wunsch, Karsten Conrad, Dirk Roggenbuck

**Affiliations:** 1 Medipan GmbH, Dahlewitz, Germany; 2 Department of Medicine II, Saarland University Hospital, Saarland University, Homburg/Saar, Germany; 3 Liver and Internal Medicine Unit, Medical University of Warsaw, Warsaw, Poland; 4 Division of Gastroenterology, Department of Internal Medicine, Faculty of Medicine, University of Debrecen, Debrecen, Hungary; 5 Institute of Biotechnology, Faculty Environment and Natural Sciences, Brandenburg University of Technology Cottbus-Senftenberg, Senftenberg, Germany; 6 Department of Laboratory Medicine, Peking University People’s Hospital, Beijing, China; 7 Translational Medicine Group, Pomeranian Medical University, Szczecin, Poland; 8 Institute of Immunology, Technical University Dresden, Dresden, Germany; 9 Faculty of Health Sciences, Joint Faculty of the Brandenburg University of Technology Cottbus-Senftenberg, the Brandenburg Medical School Theodor Fontane and the University of Potsdam, Universitätsplatz 1, 01968 Senftenberg, Germany

**Keywords:** Primary sclerosing cholangitis, Liver cirrhosis, Cholangiocarcinoma, Immunoglobulin A, Inflammatory bowel disease, Crohn’s disease, Ulcerative colitis, Microbiota, Glycoprotein 2

## Abstract

Unlike in other autoimmune liver diseases such as autoimmune hepatitis and primary biliary cholangitis, the role and nature of autoantigenic targets in primary sclerosing cholangitis (PSC), a progressive, chronic, immune-mediated, life threatening, genetically predisposed, cholestatic liver illness, is poorly elucidated. Although anti-neutrophil cytoplasmic antibodies (ANCA) have been associated with the occurrence of PSC, their corresponding targets have not yet been identified entirely. Genome-wide association studies revealed a significant number of immune-related and even disease-modifying susceptibility loci for PSC. However, these loci did not allow discerning a clear autoimmune pattern nor do the therapy options and the male gender preponderance in PSC support a pathogenic role of autoimmune responses. Nevertheless, PSC is characterized by the co-occurrence of inflammatory bowel diseases (IBD) demonstrating autoimmune responses. The identification of novel autoantigenic targets in IBD such as the major zymogen granule membrane glycoprotein 2 (GP2) or the appearance of proteinase 3 (PR3) autoantibodies (autoAbs) have refocused the interest on a putative association of loss of tolerance with the IBD phenotype and consequently with the PSC phenotype. Not surprisingly, the report of an association between GP2 IgA autoAbs and disease severity in patients with PSC gave a new impetus to autoAb research for autoimmune liver diseases. It might usher in a new era of serological research in this field. The mucosal loss of tolerance against the microbiota-sensing GP2 modulating innate and adaptive intestinal immunity and its putative role in the pathogenesis of PSC will be elaborated in this review. Furthermore, other potential PSC-related autoantigenic targets such as the neutrophil PR3 will be discussed. GP2 IgA may represent a group of new pathogenic antibodies, which share characteristics of both type 2 and 3 of antibody-mediated hypersensitive reactions according to Coombs and Gell.

## The putative impact of autoimmunity in PSC

Primary sclerosing cholangitis (PSC) is a chronic immune-mediated, life threatening, genetically predisposed liver disease with a largely unknown pathogenesis [1]. The prevalence of PSC is estimated at up to 16.2 per 100,000 individuals and is increasing [2–4].

Primary sclerosing cholangitis is characterized by cholestasis due to inflammatory and fibrotic changes in large bile ducts. The disease has a progressive course eventually resulting in biliary fibrosis and liver cirrhosis in a proportion of cases finally. Moreover, patients with PSC have an increased risk to develop hepatobiliary (most frequently cholangiocarcinoma [CCA]) and extrahepatic neoplasia independent of the duration and activity of the disease. To date there is no causative treatment available and liver transplantation remains the only curative therapy [1, 4].

The impact of autoimmune responses in the pathophysiology of PSC is still largely unknown. Neither therapeutic options nor male gender preponderance in PSC are indicative for a significant pathogenic role of autoimmune responses. Indeed, the administration of immunosuppressive drugs for PSC patients is at least controversial and rarely recommended [5, 6]. Only in the case of an overlap with autoimmune hepatitis (AIH), immunosuppressive therapy is considered [7].

Cellular immune responses might be involved in the pathophysiology of PSC [8]. However, in contrast to primary biliary cholangitis (PBC), regulatory follicular T helper cells in patients with PSC appear to have less impact on the cholestatic pathophysiology [9].

The currently known susceptibility loci do also not allow to discern a clear autoimmune pattern, though the association with distinct HLA haplotypes suggests an involvement of acquired immune responses [10]. In particular, the association of such susceptibility loci as CD28, IL2 and IL2RA (alpha subunit of the high-affinity IL2 receptor) with PSC risk lends credit to the assumption that the T lymphocyte-focused IL-2 pathway plays a putative role in the pathogenesis of PSC. In this context, an enterohepatic circulation of lymphocytes primed in the gut and supported by a pathological co-expression of adhesion molecules (vascular adhesion protein 1, mucosal addressin cell adhesion molecule 1) in the liver and gut of patients with PSC and IBD has been suggested [11, 12].

Of interest, PSC is associated with the co-occurrence of inflammatory bowel diseases (IBD) which are known to be affected by autoimmune responses [13, 14]. Up to 70% of PSC cases show concomitant IBD, especially the distinct phenotype of ulcerative colitis (UC) [2]. This appears to be a unique feature among autoimmune liver diseases. Patients with PBC rarely suffer from IBD whereas only approximately 8% of AIH presenting as overlap with PSC show concomitant IBD [15].

The two main clinical entities of IBD are Crohn’s disease (CD) and UC, both relapsing systemic inflammatory illnesses [13, 14, 16, 17]. As one of the most frequently diagnosed IBD in Caucasians (up to 322 per 100,000 individuals in Europe), CD can affect any segment of the digestive tract and is characterized by transmural inflammation [18, 19]. Prevalence rates of UC, which demonstrates superficial mucosal ulcerations restricted to the colon, may even reach 505 per 100,000 individuals [20, 21].

Primary sclerosing cholangitis patients with concomitant UC demonstrated an elevated risk of liver disease progression [5]. Conversely, CD and IBD absence appeared to confer prognostic favor in PSC and a lower risk to develop adverse effects. Of note, with regard to location of disease, CD in PSC seems to involve the colon and rarely the small bowel alone. The onset of IBD symptoms in PSC is variable and a trend towards IBD preceding PSC with a milder but more extensive intestinal inflammation compared to classical CD and UC has been observed [22].

Akin to PSC, there is no causal treatment for IBD and the illness may progress with repeated flare-ups to therapy or digestive failure requiring surgical intervention [23, 24]. Moreover, IBD is also associated with an increased risk of various intestinal and extraintestinal malignancies occurring already in adolescents and young adults [25–27]. Most likely owing to environmental factors such as Western lifestyle, diet, and industrialization, an alarming rise in the incidence and prevalence of IBD is noticed worldwide whereas comorbidity with PSC in IBD in common is underestimated [2, 19].

To date, 23 susceptibility loci have been identified by genome-wide association studies (GWAS) for PSC [28]. In contrast, recent GWAS for CD and UC revealed 163 IBD-associated loci with a certain overlap for both entities [29, 30]. These regions contain candidate genes for a variety of functions such as autophagy, microbe recognition, lymphocyte signaling, response to endoplasmic reticulum stress, cytokine signaling and others. Regarding the comparison of susceptibility loci of PSC and concomitant IBD, significant associations to the same region of the genome appeared not always to be driven by a common causal variant. Moreover, genome-wide comparisons of PSC with CD and UC showed that the comorbid gastrointestinal inflammation seen in the majority of PSC patients cannot be fully explained by a shared genetic risk [28]. Thus, PSC and comorbid PSC/IBD phenotypes might be different entities at least in terms of the genetic background. Consequently, autoimmune responses in PSC and PSC with concomitant IBD could evolve on a different genetic background.

## Humoral autoimmune responses in PSC

Given the close association of PSC with IBD, the discovery of distinct autoantigenic targets in IBD and the use of the corresponding autoantibodies (autoAbs) for IBD serology renewed the interest in humoral autoimmune responses in PSC [16, 17]. Despite numerous attempts, however, no autoantigenic targets could be identified in PSC for a long time. Moreover, PSC-specific autoAbs determined by immunofluorescence assay (IFA) did not correlate with severity or phenotype of disease. Nevertheless, similar to IBD, research on the presumed impact of autoimmune responses in PSC was mainly triggered by autoAb studies [31].

The occurrence of autoAbs in IBD was first shown for UC by revealing autoreactivity against intestinal cellular antigens in the late 1950s [32–34]. Later, humoral autoreactivity against neutrophil targets was reported in patients with UC and interestingly PSC by the detection of antineutrophil cytoplasmic autoAbs (ANCA) with immunofluorescence assay (IFA) [35–37].

In CD, the first report on humoral autoimmunity referred to the occurrence of autoAbs against buccal mucosa cells [38] and, later on, to exocrine pancreas (PAb) ascertained by IFA [39, 40]. Whereas autoimmunity in UC could be linked to the site of colonic inflammation, the occurrence of PAb in CD remained an unsolved enigma until recently. Though there is a certain correlation of PAb with idiopathic chronic pancreatitis as extraintestinal complication, the frequency thereof is virtually the same in PAb-positive and PAb-negative patients with CD [41–44]. Thus, the role of a loss of tolerance to exocrine glands of the oral cavity and in particular to the exocrine pancreas is difficult to explain in the context of inflammatory changes in the intestine [45].

In PSC, humoral autoimmunity in the form of autoAbs to several targets including biliary epithelial targets was reported [31, 46]. However, only atypical ANCA detected by IFA or more precisely peripheral anti-neutrophil nuclear autoAbs (p-ANNA) recognizing a putative 50 kDa protein of the nuclear membrane have been considered diagnostic for PSC [47–50]. The abbreviation p-ANNA should not be confused with antineuronal nuclear antibody (Ab) used in the context of the serology of paraneoplastic neuronal autoimmunity.

Until lately, there have been only few reports that the above mentioned autoAbs in patients with IBD and PSC are correlated with clinical parameters or even the phenotype of the disease. Consequently, they have not widely been employed in clinical routine. Thus, the identification of the respective autoantigenic targets could help in shedding light on the role of humoral autoimmunity in IBD and PSC. This would furthermore enable the development of clinically useful tools for the diagnosis thereof.

### Identification of autoantigenic targets in CD

Autoimmune processes have been considered to play an active role in disease development and to modulate inflammatory processes in CD [51]. Therefore, the recent identification of humoral autoantigens in CD provided a new impetus for this hypothesis. Moreover, the subsequent detection of these new autoAbs and their association with the phenotype and severity of PSC was a remarkable finding [52].

Only 25 years after the first report on PAb in patients with CD and numerous unsuccessful attempts by several research groups, the corresponding molecular autoantigenic targets could be discovered [40, 53–57]. Lastly, glycoprotein 2 (GP2) was independently described by two groups as autoantigenic target of PAb associated with CD [58, 59]. Apart from GP2, Stöcker’s group discovered CUB/zona pellucida like domain-containing protein 1 (CUZD1) as a second antigenic target of PAb [59]. Interestingly, PAb stain different exocrine pancreatic moieties in IFA and two types of PAb are reported (type I; extracellular drop-like staining of the acinar lumen; type II: speckled cytoplasmic staining of acinar cells) [60, 61]. As the majority of type II PAb-positive sera revealed concomitant PAb I reactivity, these two IFA patterns could also be the result of just one autoantigenic target such as GP2 [45, 62].

Altogether, the identification of GP2 and CUZD1 as autoantigenic targets in CD ushered in a new era in IBD serology and triggered an impressive number of clinical studies investigating the potential role of the respective autoAbs in the differential diagnosis of IBD [63, 64].

Already two meta-analyses encompassing 17 and 15 serological studies have been reported to date [65, 66]. For GP2 autoAbs they revealed pooled diagnostic sensitivities of 24% and 20% as well as pooled diagnostic specificities of 96% and 93%, respectively. In comparison to the established Ab to *Saccharomyces cerevisiae* (ASCA) in CD serology, autoAbs to GP2 demonstrated a remarkable specificity allowing even the discrimination of intestinal diseases with similar clinical symptoms such as intestinal tuberculosis and Behcet’s disease [67]. This is of diagnostic importance, since these illnesses are difficult to discriminate from CD by endoscopic methods, which are still the basic tools for gastroenterologists in the context of this differential diagnosis. Of note, patients double positive for GP2 autoAb and ASCA showed a 100% specificity regarding the differentiation of CD from UC underscoring the usefulness of autoAb/Ab profiling in the differential diagnosis of IBD [68].

The moderate sensitivity of GP2 autoAb appears to limit its use as diagnostic marker for CD [66]. However, significant associations of CD-specific autoAbs could be established with the severity and phenotype of disease stratified in accordance with the Montreal classification by different studies [64]. Thus, GP2 autoAbs are linked with onset of disease at younger age (A1), ileal/ileocolonic inflammation (L1/L3) and a more severe course of disease (B2/B3). Regarding the latter, GP2 autoAbs are correlated with progressive strictures and need for surgery in CD [69, 70]. Altogether, given the variability of the CD phenotype, GP2 autoAb appears to be a valuable marker for a severe CD with fibrotic manifestations. Moreover, it could aid in the differentiation of recently proposed clinical subtypes of CD [71]. In contrast to fecal calprotectin, an established surrogate marker of active intestinal inflammation in IBD, GP2 autoAb levels do not correlate with disease activity [72]. However, GP2 autoAb appears to be linked with the chronicity of inflammation as shown for the occurrence of GP2 IgA in celiac disease [73–75]. Similar to celiac disease-specific IgA reactive with transglutaminase or deamidated gliadin, GP2 IgA levels were significantly reduced and eventually became negative after the initiation of a gluten-free diet as causal therapy [73]. Thus, GP2 IgA could be a candidate for a marker for the successful treatment of CD from an immunological point of view.

Similar studies for autoAbs to CUZD1 supporting an association with disease phenotypes (early onset and perianal disease) have been scarce or have not shown a significant correlation [76–78]. Papp et al. [52] reported GP2 autoAbs as an independent predictor of surgery whereas autoAbs to CUZD1 predicted perianal disease in the only prospective study available to date. For the first time, GP2 and CUZD1 autoAbs were associated with the co-occurrence of PSC and cutaneous manifestations in this study, respectively [52]. Michales et al. [77] deploying the same assay techniques, however, could not confirm significant associations with extraintestinal manifestations. Nevertheless, the prospective study by Papp et al. was the starting point for the investigation of GP2 as an antigenic target in PSC.

Remarkably, GP2 autoAb occurrence could be linked with de novo CD in patients suffering from severe UC with pouchitis after colectomy and ileal pouch anal anastomosis (IPAA) [79, 80]. This underscores a close link of the occurrence of GP2 autoAbs with the change of microbiota within the pathophysiology of CD-like symptoms in a formerly UC-driven inflammatory environment. Further evidence for an infectious origin with related changes of the microbiota comes from studies on the animal model of CD in ruminants with *Mycobacterium avium* induced paratuberculosis [81–83]. GP2 appears to be the only specific target of PAb linked with the loss of tolerance seen in this animal model.

### Identification of autoantigenic targets in UC

In contrast to CD, ANCA to unknown neutrophil targets were already reported in the 1980s as serological markers of PSC and UC [35, 37]. However, the attempts to discover the corresponding autoantigenic targets of these ANCA or of UC-specific autoAb to intestinal goblet cells did not provide consistent results [84, 85].

Teegen et al. [85] have reported DNA-bound lactoferrin as the main autoantigenic target of ANCA in patients with UC, however, the finding has not yet been confirmed by others. Nonetheless, these autoAbs to a neutrophil target in combination with DNA showed a high prevalence in patients with UC recently [86]. In this extensive prospective evaluation of autoAbs and antimicrobial Abs in patients with UC, only ASCA IgA could be identified as an independent predictor of long-term immunosuppressive therapy with regard to the clinical phenotype association of UC-specific antibodies [86]. This was a surprising finding, as ASCA was commonly reported to be specific for CD.

Moreover, the identification of a colon specific 40 kDa murine protein linked to UC could be reproduced in humans, but the sequence analysis of this target failed to match it with a particular molecule [87].

Hence, the independent reports of autoAbs to proteinase 3 (PR3), a neutrophil target, by sensitive bead-based chemiluminescence and fluorescence techniques in patients with UC provided a new diagnostic option for the differential diagnosis of IBD [88–91]. Despite the excellent discrimination of UC from CD by PR3-autoAb positivity and the association with more extensive inflammation in UC, the finding raised a controversial discussion. PR3 autoAb, also referred to as PR3-ANCA, has been considered a specific marker of granulomatosis with polyangiitis (GPA) formerly known as Wegener’s granulomatosis [92]. Moreover, the majority of PR3 autoAb-positive sera of patients with GPA appear to demonstrate a classical cytoplasmic staining pattern on neutrophils (cANCA) by IFA. This finding is not in line with the atypical perinuclear ANCA or perinuclear antineutrophil nuclear antibody (p-ANNA) pattern commonly determined with sera of UC patients.

Nevertheless, this intriguing finding warrants further clinical evaluation to elucidate the putative autoantigenic role of PR3 in UC and its possible link with the pathophysiology in GPA. It provides further evidence, however, for a potential role for neutrophils in the pathophysiology of IBD as does the report of DNA-dependent lactoferrin autoAbs. Due to the high prevalence of concomitant UC in patients with PSC, the question of a potential role of PR3 as an autoantigenic target in PSC began to appear on the horizon.

### Autoantigenic targets in PSC—lessons from humoral autoimmunity in IBD

At the beginning of the millennium, IgG autoAbs recognizing biliary epithelial cells have been shown to be specific for PSC and in this context combine adaptive and innate immune responses [46, 93]. However, despite their reported potentially pathophysiological implications, to date the autoimmune targets of these autoAbs have not been identified. Nevertheless, this is another important finding, which underscores the involvement of loss of tolerance in the pathogenesis of PSC.

Of note, all other relevant PSC-specific autoAbs appear to recognize non-biliary targets [48]. Thus, the search for autoantigenic targets in PSC was increasingly based on serological studies with IBD patients.

Akin to UC, atypical ANCA or more precisely p-ANNA have been one of the most debated diagnostic markers in PSC [48, 94]. After the promising discovery of a neutrophil, nuclear envelope-target molecule for p-ANNA, Terjung et al. [95] identified beta-tubulin isotype 5 as a novel ANCA autoantigen in PSC. This target shares a high structural homology with the microbial cell division protein FtsZ. Unfortunately, this finding could not be corroborated in other studies [96]. Moreover, all these autoAbs and their corresponding targets previously reported in patients with PSC do not seem to be directly associated with the clinical symptoms of the illness [31, 97].

Thus, the report on PR3 autoAbs detected by a sensitive chemiluminescence assay in patients with PSC and its correlation with elevated liver enzymes, particularly with alkaline phosphatase, renewed the interest in potential ANCA targets in PSC [98]. As outlined above in the context of UC, the pathophysiological role of a tolerance loss to PR3 in PSC is controversial due to the established role of PR3 as a specific autoantigenic target in GPA. Of note, a recent case report of a female patient with suspected hepatically localized GPA could be an illustrating example in this context [99]. The first diagnostic assumption focused on vasculitis and particularly GPA as the cause of clinical symptoms due to the PR3 autoAb positivity. However, because of the lack of both typical GPA symptoms and involvement of other organs on the one hand and elevated alkaline phosphatase levels on the other hand, this diagnosis was not confirmed [99]. In contrast, the PR3 autoAb positivity along with the cholestatic symptoms rather suggested the presence of PSC with concomitant loss of tolerance to neutrophil components and not GPA.

Besides the association of PR3 autoAb with the PSC phenotype, another groundbreaking finding came into the spotlight of autoimmune research in PSC. The first report in the year 2015 on the association of GP2 autoAb with concomitant PSC in patients with UC triggered several studies on the role of loss of tolerance to GP2 in PSC [52]. This finding could be corroborated by the same group in an elegant prospective study in patients with UC demonstrating a correlation of GP2 IgA and not IgG with UC and concomitant PSC [86]. Since GP2 autoAb has been confirmed as a highly specific serological marker for CD, this indicates that this autoAb is associated with PSC and not with UC. It also provides an explanation for the “false-positive” UC cases for the GP2 autoAb. This assumption was supported by a seminal paper investigating two independent European PSC cohorts [100]. A remarkable prevalence of around 50% was shown for GP2 IgA in patients with PSC, secondary cholangitis and most intriguingly CCA. Moreover, GP2 IgA was significantly associated with disease severity and poor patient survival in this study [100]. The latter association was mainly due to CCA and its corresponding high mortality rate. Whereas Tornai et al. also reported a weak but significant association of CUZD1 IgA with PSC in UC patients, CUZD1 IgG as well as IgA did not show any correlation with the disease phenotype in the two other European PSC cohorts [100]. Altogether, this was the first report linking an autoAb with the phenotype and occurrence of CCA in patients with PSC. More interesting is the fact that IgA and not IgG reactivity to GP2 was responsible for this correlation. This hints at an involvement of the mucosa-associated lymphoid tissue (MALT). Recently, Tornai et al. [101] confirmed the association of GP2 IgA with the severity of PSC in a prospective study demonstrating a significant correlation with shorter transplant-free survival for GP2 autoAbs. GP2 IgA was the only independent predictor for liver transplantation after adjusting for Mayo risk score with a hazard ratio of 4.69. Another intriguing finding was the correlation of GP2 IgA occurrence with elevated levels of secretory IgA (three times the normal value) which could be a sign of an increased IgA secretion by epithelia and/or reabsorption of IgA from mucosal surfaces. In contrast, this phenomenon was not revealed for all investigated microbial Abs and autoAbs including ANCA [101].

In total, autoAbs to four isoforms of GP2 have been reported in patients with IBD [102, 103]. These GP2 isoforms encompass two larger and two smaller isoforms whereby the large and small isoforms differ in only three amino acids (valine–proline–arginine) [64]. Intriguingly, autoAbs to all four GP2 isoforms, which were expressed as glycosylphosphatidylinositol (GPI)-anchored membrane molecules in a human cell line, could be determined by IFA in patients with PSC from four European university hospitals [104]. Combined testing for IgA to GP2 isoforms 1 (large isoform) and 4 (small isoform) was superior to the analysis of autoAbs to just one GP2 isoform and resulted in a sensitivity of 66% in the 212 patients with PSC [104]. AutoAbs to GP2 isoforms 1 and 4 were independent risk predictors for the severity of disease (occurrence of cirrhosis) after adjusting for age and gender [104].

In summary, GP2 seems to function as a unique autoantigenic target in CD and in PSC. Given the close link with disease severity and carcinogenesis, IgA to GP2 and its isoforms have the potential to become accepted as pathognomonic and predictive for PSC. It remains to be shown what additional diagnostic or even prognostic role PR3 autoAbs can play in the serology of PSC.

## GP2 as an autoantigenic target in PSC—indication of microbial involvement?

The novel autoantigenic target in PSC, GP2, is not organ-specific nor is the PR3-AutoAb. In this context it is interesting to note that the much debated controversy on the expression of CD-specific autoantigenic targets in extraintestinal organs (pancreas or oral cavity) and not at the site of inflammation in the gut could only be overcome for GP2 [58, 105]. Elevated transcription of GP2 mRNA and translation of GP2 being a 78 kDa GPI-anchored molecule with N-linked carbohydrates in intestinal biopsy samples was only shown for patients with CD in contrast to patients with UC [58]. Unlike for GP2, evidence for the expression of the other PAb target CUZD1 in the intestine and a possible immunomodulating role thereof is still lacking [106].

In earlier studies, GP2 was identified as the major pancreatic zymogen granule membrane protein with an assumed but non-confirmed role in zymogen granula formation [107–112]. Thus, the discovery of GP2 as microbiome-sensing receptor for particularly FimH-positive bacteria on microfold (M) cells of the intestinal follicle-associated epithelium (FAE) fundamentally changed the understanding of GP2’s physiological role [113, 114]. Peyer’s patches (PP) harbouring M cells and located in the epithelium covering MALT of the small intestine play a pivotal role in intestinal immune responses [115]. Along with the Ets transcription factor Spi-B, GP2 is a specific marker of mature M cells characterized by high up-take activity of luminal components [116, 117]. Active inflammation in CD has been shown in intestinal PP which are even regarded as potential sites of the inflammatory onset [118–120]. There is growing evidence that intestinal dysbiosis in connection with an impaired intestinal immune response has a relevance to the development of autoimmune disorders [115]. In line with this assumption, an elevated risk for the onset of CD after gastrointestinal infections has been reported [121].

In this context, GP2’s modulating role of innate and adaptive immune responses by sensing microbiota and regulating intestinal anti-microbial immune responses is quite remarkable [122–126]. The unique expression profile of GP2 in mucous glands of the upper digestive tract and pancreas as well as on intestinal M cells suggests a physiological balance in regard to the binding of FimH-positive bacteria by secreted (soluble) and membrane-bound GP2 in the gut [62]. High levels of adhesive *E. coli* which can target human PP via long polar fimbriae and point mutations in their FimH amino acid sequences were linked with CD [127–129]. Interestingly, a reduced GP2 presence on microbial surfaces in the intestine of CD patients has been reported, indicating a disturbed balance between soluble and membrane-bound variants of GP2 in CD inflammation [51, 62]. Furthermore, the association of loss of tolerance to GP2 with the appearance of de novo CD-like inflammation in patients with pouchitis supports the involvement of a disturbed interaction of GP2 with microbiota in the onset of CD [79, 130]. This finding is remarkable since an initial UC-specific inflammatory environment switched to a CD-like one with autoimmunity to GP2 occurring presumably due to the new microbiota composition in the pouch after colon resection. This alludes to a pathogenic role of GP2 autoAbs particularly of the IgA isotype. Thus, an inadequate immune response to an infectious agent or dysbiotic microbiota may trigger inflammatory processes involving autoimmunity against M-cell receptors like GP2.

With regard to liver autoimmunity, loss of tolerance to similar surface receptors such as the hepatocyte-specific asialoglycoprotein receptor interacting also with potential pathogens or their components has been demonstrated in patients with AIH and PSC recently [131, 132]. Alike ASGPR autoAbs in AIH, GP2 autoAbs are closely associated with the severity of disease in patients with PSC. However, hitherto there is no report on the presence of GP2 in the biliary tract. Thus, autoimmune processes in the FAE of the gut could be responsible for triggering or perpetuating pathophysiological changes in the biliary tract in the context of the extensively discussed gut-liver axis.

## Putative role of GP2 IgA in the pathophysiology of PSC

**Fig. 1:**
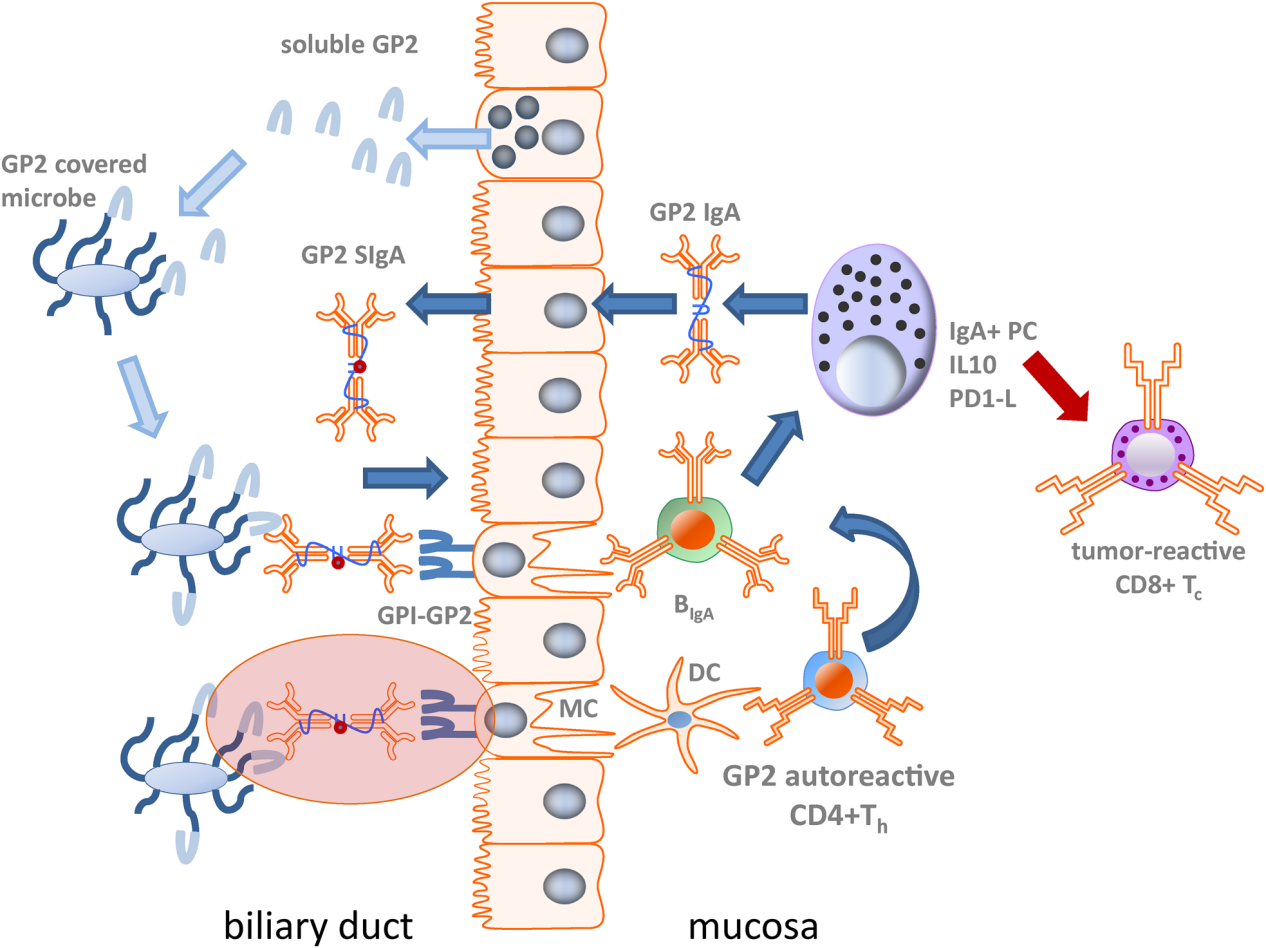
Putative pathophysiological role of mucosal autoimmunity to GP2 in PSC. After mucosal loss of tolerance to glycoprotein 2 (GP2), GP2 IgA is actively secreted by biliary epithelial cells into bile as GP2 secretory IgA (SIgA). Simultaneously, GP2 is shed from exocrine biliary cells along with secretions into the bile. GP2 specifically interacts with putative FimH-positive microbes (FimH+) and binds to GP2 SIgA. The latter could link the recognized microbe with GP2, membrane-bound by a glycosylphosphatidylinositol (GPI) anchor to the apical surface of biliary or intestinal microfold cells of the follicle-associated epithelium. M cells transcytose the GP2-microbe complex and present it to antigen-presenting cells such as IgA-positive (IgA+) B cells or dendritic cells (DC) of the underlying mucosa-associated immune system. Subsequently, IgA+ B cells including GP2-reactive cells are triggered which differentiate directly or by CD4-positive T-helper cells (CD4+ T_h_) assistance into immunosuppressive IgA-secreting plasma cells (IgA+ PC) shedding interleukin 10 (IL10) and programmed cell-death 1 ligand (PD1-L). The latter two are considered suppressors of tumor-surveillance components such as cytotoxic CD8− positive T cells (CD8+ T_c_). Taken together, this hypothetical vicious cycle suggests a new pathogenic mechanism for antibodies encompassing features of types 2 and 3 of hypersensitive immune reactions in accordance with the classification of Coombs and Gell by involving microbiota (coloured oval area). (Adapted according to [62])

Given the association of both GP2 IgG and IgA to the fibrostenosing subtype and the severity of CD, it is quite remarkable that only IgA to GP2 has been linked with the clinical phenotype and severity in PSC. Of note, dimeric GP2 IgA like most of the IgA secreted by mucosal plasma cells could be actively transported by epithelial cells onto mucosal surfaces as has been shown for GP2 IgA in pouchitis patients with de novo CD [79]. Thus, GP2 IgA might interact with bacteria coated with GP2 of pancreatic origin in the intestine and further cross-link it with the GPI-anchored GP2 on M cells due to its dimeric nature. That would support the up-take of particularly FimH-positive microbes by M cells and could enhance the transcytosis of potentially pathogenic adherent bacteria and subsequently inflammatory processes in the intestinal mucosa [62]. If such a vicious cycle of perpetuating inflammation could be established for the biliary tract in PSC remains to be shown (Fig. 1). It would require the secretion of GP2 into the bile by exocrine glands such as periductal mucous glands and the presence of pathogenic microbes in the bile. Oral, respiratory and genital mucus glands have been demonstrated to secret GP2 in mice apart from the pancreas as the main source of intestinal GP2 [105]. GP2 was also identified as a major component of pancreatic intraductal plugs in chronic pancreatitis which resembled hyaline casts containing uromodulin, a renal GP2 homolog, in the urinary tract [133–135]. Given the significant correlation of loss of mucosal tolerance to GP2 to the pathophysiology of obstructive fibrotic changes in the biliary tract, the presence of GP2 in bile and its participation in gallstone formation would support a pathogenic role of GP2 IgA.

There is growing evidence to suggest that the biliary tract is not a sterile environment as previously thought [136, 137]. A possible involvement of the microbiota in PSC could also be supported by the identification of a genetic polymorphism linked with PSC as well as CD that generated a dysfunctional fucusyltransferase-2 (FUT2) [138, 139]. These FUT2 variants appear to affect carbohydrate metabolism in the gut and consequently influence the microbiome. In this context, a reduced diversity particularly in the Firmicutes and Bacteriodetes phyla has been found in IBD [140–144]. A diminished content of the firmicute *Faecalibacterium prausnitzii* was linked with an elevated risk of postoperative recurrence of ileal CD [145]. Butyrate producing bacteria seem to have an anti-inflammatory effect in CD. In PSC, clinical trials demonstrated a beneficial effect of antibiosis on liver enzyme levels [146]. The aetiology of PSC does not appear to be associated with specific changes in biliary microbial communities [136]. However, the genus *Streptococcus* is considered to play a putative role in the progression of PSC.

Finally, an elevated secretion and re-absorption of secretory IgA was reported in patients with PSC [52]. The majority of GP2 IgA in the serum of CD patients bears a secretory piece [101]. This indicates that this particular GP2 IgA was secreted onto mucosal surfaces and later re-adsorbed. Indeed, GP2 IgA was detected in faeces of patients with CD-like inflammation which supports the active secretion of GP2 IgA into the intestinal lumen in such conditions [79, 130].

Hence, it is tempting to speculate that GP2 as microbiota-sensing receptor and the ensuing mucosal loss of tolerance are not only involved in the pathophysiology of CD, but also in that of PSC (Fig. 1). It would provide the basis for a new pathogenic mechanism of antibody reaction which encompasses features of second and third types of Coombs and Gell classification of hypersensitive immune responses but requires the involvement of the microbiota [147]. The reports of elevated GP2 IgA in patients with active celiac disease and particularly refractory variants thereof further support such assumption [73–75].

In CD, GP2 IgA aside from GP2 IgG has been linked with fibrotic changes in several studies whereas only GP2 IgA was associated with severity in PSC (Table 1). Concentric fibrosis of the intra- and extrahepatic bile tracts is the pathophysiological hallmark of PSC. In spite of the impressive number of clinical studies on the link of GP2 autoAbs with fibrosis, it remains to be demonstrated if the autoimmune hypothesis on the induction or perpetuation of mucosal inflammation in PSC and CD holds true.

**Table 1:** Clinical studies demonstrating an association of IgG and IgA to glycoprotein 2 (GP2) with fibrosis as well as disease severity in Crohn’s disease (CD), ulcerative colitis (UC) with ileal pouch anal anastomosis (IPAA) and primary sclerosing cholangitis (PSC)

Illness	Study design	Number of patients	Assay technique	GP2 autoantibody isotype	Associations with clinical phenotype	Reference, year
CD	Retrospective	169	ELISA	IgG	A1, B2/PD, B3(−), L2(−), L3	Bogdanos et al. 2012 [69]
				IgA	L2(−)	
				IgG/IgA	A1, B2/PD, B3(−), L2(−), L3	
UC with IPAA	Retrospective	26	ELISA	IgG	CD of the pouch	Werner et al. 2013 [79]
				IgA	CD of the pouch	
				IgG/IgA	ND	
CD	Retrospective	303	ELISA	IgG	B2, NS	Rieder et al. 2013 [152]
				IgA	B2, NS	
				IgG/IgA	ND	
CD	Retrospective	86	ELISA	IgG	B2/B3	Kohoutova et al. 2014 [153]
				IgA	B2/B3, B3	
CD	Retrospective	323	ELISA	IgG	DD, L2(−), L3	Pavlidis et al. 2014 [68]
				IgA	A1	
				IgG/IgA	A3(−), B2, B1(−), DD, L3	
CD	Retrospective	224	IFA	IgG	ND	Michaelis et al. 2015 [77]
				IgA	ND	
				IgG/IgA	B2, DD, L2(−)	
CD	Prospective	271	ELISA	IgG	B2/B3, NS	Papp et al. 2015 [52]
				IgA	B2/B3, NS	
				IgG/IgA	B2/B3, NS	
			IFA	IgG	L1	
				IgA	B3, L1	
				IgG/IgA	–	
CD	Retrospective	212	IFA	IgG	A1, B2, L2(−)	Pavlidis et al. 2016 [76]
				IgA	–	
				IgG/IgA	–	
CD	Retrospective	303	ELISA	IgG	NS, stenosis	Degenhardt et al. 2016 [65]
				IgA	NS, stenosis	
				IgG/IgA	ND	
CD	Retrospective	164	ELISA	IgG	B2/3, NS	Röber et al. 2017 [103]
				IgA	B2/3, NS, PD	
				IgG/IgA	ND	
PSC	Prospective	218 (138,180)	IFA	IgG	–	Jendrek et al. 2017 [87]
				IgA	Poor survival, CCA	
				IgG/IgA	ND	
CD	Retrospective	171	ELISA	IgG	A3(−), B3, PD	Zhang et al. 2018 [61]
				IgA	L1, L3(−), B3	
				IgG/IgA	A3(−), L1, B3, PD	
UC with IPAA	Prospective	177	ELISA	IgG	CD of the pouch	Cummings et al. 2018 [80]
				IgA	CD of the pouch	
				IgG/IgA	ND	
PSC	Retrospective	212 (23,30,83,76)	IFA	IgG	–	Sowa et al. 2018 [104]
				IgA	Cirrhosis	
				IgG/IgA	–	
PSC	Prospective	65	IFA	IgG	–	Tornai et al. 2018 [101]
				IgA	Predictor of shorter transplant-free survival	
				IgG/IgA	–	
PSC	Prospective	338	ELISA	IgG	–	Wunsch et al. 2019 (13th Dresden Symposium on Autoantibodies, September 2019)
				IgA	Predictor of shorter transplant-free survival and CCA	
				IgG/IgA	–	

Montreal classification of CD in accordance to age, A1: < 17 years, A2: 17–40 years, A3: > 40 years, behaviour of disease, B1: non-stricturing/non-penetrating, B2: stricturing, B3: penetrating, location of disease, L1: ileal, L2: colonic, L3: ileocolonic, L4 upper gastrointestinal tract

(−), significantly negative association; CCA, cholangiocarcinoma; DD, disease duration; ND, not determined; PD, perianal disease; NS, need for resective surgery

The close association of GP2 IgA with tumorigenesis in PSC is another intriguing point regarding a putative pathophysiological role of GP2 IgA. The two main precursor lesions of cholangiocarcinoma are biliary intraepithelial neoplasia and intraductal papillary neoplasm of the bile duct [148].

In general, the intestinal mucosa harbours the largest population of immunoglobulin-secreting plasma cells in humans, shedding daily several grams of IgA. This exceeds the production of all other immunoglobulin subtypes in the human body [149]. IgA-positive plasma cells have been shown to secret immunosuppressive interleukins (IL) such as IL10 and programmed cell death 1 ligand (PD1L) [150]. Plasma cells in the biliary mucosa have been considered as the likely source of most of the locally synthesized IgA that is secreted into human hepatic bile [151]. Remarkably, non-alcoholic fatty liver disease in mice and humans is linked with the accumulation of liver-resident IgA-secreting cells which express PD-L1 and IL10 [150]. These cells can directly suppress liver cytotoxic CD8+ T lymphocytes and, thus, foster the occurrence of hepatocellular carcinoma. A similar scenario could be assumed for neoplastic changes in PSC with autoreactive IgA positive plasma cells as a key player.

Thus, in terms of a putative pathogenic role of the mucosal loss of tolerance to GP2 in PSC, the above mentioned findings give rise to the following questions:

1. Does secretory GP2 IgA enhance the up-take of pathogenic microbes which in turn further trigger the generation of plasma cells secreting IgA autoAbs?
2. Is GP2 secreted into the bile by exocrine glands?
3. Is there a specific microbe in the bile which can interact with GP2?
4. Are GP2 IgA-positive plasma cells abundant in the biliary mucosa in patients with PSC?
5. Do GP2 IgA-positive plasma cells have an immunosuppressive phenotype fostering neoplasia and impacting the immune surveillance of tumor cells?

## Summary

The putative pathogenic role of PSC-specific (auto)Abs has not yet been addressed by appropriate studies. There is an urgent need to shed a light on the yet unresolved pathophysiological role of autoimmunity in PSC, associated IBD and finally carcinogenesis to provide more effective diagnostic and therapeutic strategies. We assume that mucosal autoimmunity to GP2 could be a promising candidate to demonstrate active involvement of autoimmune responses based on an intestinal or biliary dysbiosis in the pathophysiology of PSC. Glycoprotein 2 appears to be a unique autoantigenic target in PSC. Thus, GP2 has been shown (i) to be discharged by mucus glands into the digestive tract, (ii) to interact with microbiota by binding FimH-positive microbes, (iii) to be expressed as specific receptor on M cells of the FAE and (iv) to be an immunomodulating factor of innate and acquired immune responses. Consequently, the mucosal loss of tolerance to GP2 in form of GP2 IgA is a potential pathognomonic marker of PSC with predictive value that may improve the diagnosis and prognosis of the illness.

## Abbreviations

Abs: Antibodies
autoAbs: Autoantibodies
ANCA: Anti-neutrophil cytoplasmic antibodies
ASCA: Anti-*Saccharomyces cerevisiae* antibodies
AIH: Autoimmune hepatitis
CCA: Cholangiocarcinoma
CeD: Celiac disease
CD: Crohn’s disease
CUZD1: Complement subcomponents
C1r/C1s: Sea urchin Uegf protein, bone morphogenetic protein-1, zona pellucida-like domain-containing protein 1
ELISA: Enzyme-linked immunosorbent assay
FAE: Follicle-associated epithelium
GP2: Zymogen granule membrane glycoprotein 2
GPA: Granulomatosis with polyangiitis
GPI: Glycosylphosphatidylinositol
IBD: Inflammatory bowel disease
IFA: Immunofluorescence assay
IPAA: Ileal pouch anal anastomosis
MALT: Mucosa-associated lymphoid tissue
M cell: Microfold cell
PAb: Pancreatic autoantibodies
PBC: Primary biliary cholangitis
p-ANNA: Perinuclear antineutrophil nuclear antibodies
PP: Peyer’s patches
PR3: Proteinase 3
PSC: Primary sclerosing cholangitis
UC: Ulcerative colitis
